# Patient and Designer Collaboration in Cocreating Technical Innovations in a Hospital-Based Makerspace: Qualitative Interview Study

**DOI:** 10.2196/85926

**Published:** 2026-05-20

**Authors:** Jiske van der Giessen, Onno Helder, Thijs van Houwelingen

**Affiliations:** 1 Nursing Sciences, Program in Clinical Health Sciences, University Medical Center Utrecht, Utrecht University, Utrecht, The Netherlands; 2 Department of Create4Care, Erasmus MC University Medical Centre, Rotterdam, The Netherlands; 3 Research Group Technology Use in Health and Social Care, Research Centre for Healthy and Sustainable Living, University of Applied Sciences Utrecht, Heidelberglaan 7, Utrecht, 3584 CS, The Netherlands, 31 641097762

**Keywords:** patient involvement, cocreation, patient, innovation, qualitive study, nursing development, delivery of health care, communication

## Abstract

**Background:**

Hospital-based makerspaces have emerged as collaborative environments for technical innovation, where designers cocreate with health care professionals and patients to resolve specific problems experienced in practice. As end users, patients can offer unique insights that could drive the development of patient-centered health care services or research. However, cocreation with patients within a makerspace environment is still rare—representing a missed opportunity to use their insights to develop innovations that meet their needs. Patient-designer interactions are underaddressed in the literature, and a deeper understanding could enhance their effectiveness.

**Objective:**

The study aimed to explore the experiences of designers and patients (or their carers) cocreating in an academic hospital–based makerspace, using a bottom-up approach.

**Methods:**

A generic qualitative study was conducted using semistructured individual interviews based on the Sunnybrook team–based competencies and design thinking methodology. A heterogeneous sample of 12 participants was recruited from a makerspace in an academic hospital in the Netherlands, comprising 6 with a designer’s perspective and 6 with a patient’s perspective. Most participants were involved in collaborative pairs, representing both perspectives within the same innovation project. Data were analyzed thematically.

**Results:**

Four key themes emerged from the data: (1) dealing with the patient’s situations, (2) integrating different perspectives, (3) feeling valuable and useful, and (4) dynamic interplay and engagement. Collaboration with patients in this hospital-based makerspace was perceived as valuable for innovation development, benefiting from diverse perspectives. However, the degree of patient involvement varied, influenced by factors such as the patient’s health status, reachability, and interest in innovation development.

**Conclusions:**

This study highlights the complexity of developing technical innovations that involve hospitalized patients. It offers valuable insights into how both patients and designers generally viewed their collaboration in the makerspace positively. Practical recommendations for hospital-based makerspaces include respecting patient autonomy, involving patients early and consistently throughout all development phases, maintaining communication beyond the formal collaboration period, and actively seeking feedback.

## Introduction

Over the past decade, makerspaces in hospitals have contributed to a new movement in health care known as Health 4.0 [1-3]. This movement uses modern technology to improve the quality, accessibility, and cost-efficiency of health care [4,5]. Such improvement is needed to address the increasing health care demand and to enhance staff empowerment and the work environment in the face of nursing workforce shortages [6-8]. However, technical innovations designed for health care may fail to meet the needs of nurses and patients [9]. These end users themselves may need to develop innovations that align with their daily practice, and makerspaces can assist in this process.

A makerspace is a collaborative physical department equipped with prototyping facilities, including 3D printers and laser cutters [9]. Professionals such as industrial product designers, user experience designers, software engineers, and researchers are employed to facilitate innovation development. Hospital-based makerspaces aim to find suitable solutions to problems experienced in health care practice, such as poor ergonomics and time-consuming tasks. These problems are identified using a bottom-up approach, meaning that nurses and patients address their problems and cocreate with the makerspace staff to remedy the problem or develop innovations to solve them [10,11]. By recognizing patients or their carers as end users, involving them in a cocreation process offers valuable insights to address issues and develop innovations that could improve patient-centered health care [12-15].

For conceptual clarity, this study adopts the definition of cocreation proposed by Pearce et al [16], who identified 4 interrelated collaborative processes—coideation, co-design, coimplementation, and coevaluation—as the core components of cocreation in health interventions. These 4 processes align closely with the stages of design thinking: inspiration (problem identification), ideation (prototyping and testing), and implementation (diffusion of innovations). The design thinking approach is commonly used in makerspaces and provides a structured lens for examining stakeholder involvement [17].

In health care, cocreation approaches are widely promoted as a means of strengthening patient involvement, improving the relevance of interventions, and bridging the gap between knowledge generation and practice. A systematic review by Grindell et al [18] found that cocreation fosters shared understanding, responsibilities, and decision-making among stakeholders, and enables diverse types of knowledge, including patient experience, to shape innovation. Moreover, a qualitative study by Voorheis et al [19] shows that patient and public involvement does not merely inform isolated design choices but influences the broader direction and viability of an innovation, including the implementation and long-term relevance of innovations. Their study also highlights that participating in cocreation can be empowering for patients themselves. At the same time, the authors emphasize that without clear roles, sustained engagement, and supportive structures, much of this potential value remains underused. A scoping review found that patient involvement is often limited to the first 2 phases of innovation development [20,21]. Excluding patients and their carers from all phases represents a missed opportunity and could be due to a mismatch in perception of the usefulness of solutions or a lack of public awareness and guidance on how to effectively collaborate with patients in technological innovation development [22,23].

While concepts such as public and patient involvement and community-based participatory research offer valuable insights into patient involvement, they are structured around research-oriented participation models and projects focusing on the public, where larger groups of stakeholders are included. Therefore, they differ from the makerspace context, where innovation emerges through bottom-up, practice-driven problem-solving; patients are involved due to an intrinsic motivation to solve their experienced problems and are part of a smaller team within the hospital.

Although the importance of involving patients throughout innovation processes is repeatedly emphasized in the literature, empirical research examining how patients and designers actually experience cocreation within hospital-based makerspaces remains scarce. Most existing studies focus on how makerspaces function organizationally and their role, rather than on the human interactions that shape cocreation [2,3,20,24].

Understanding the collaborative experiences of collaborations between designers and patients (or their carers) is essential. Gaining insight into the barriers and facilitators of such collaboration can improve future cocreation efforts within hospital makerspaces. Therefore, this study aims to explore the experiences of designers and patients (or their carers) cocreating in an academic hospital-based makerspace, using a bottom-up approach.

## Methods

### Study Design

A generic qualitative study was performed using semistructured interviews and thematic analysis. This approach enabled an in-depth understanding of how designers and (carers of) patients perceived the cocreation and what could be learned from their experiences, rather than testing or producing a theory or explicating the essential structure of a single phenomenon [25]. The study was conducted between February 2024 and June 2024 and adhered to the COREQ (Consolidated Criteria for Reporting Qualitative Research) checklist (Checklist 1) [26].

### Setting

The study was conducted at a makerspace with a professionally equipped prototype studio, housed within an academic hospital, including a large children’s hospital in the Netherlands. The makerspace is financed through the hospital’s internal funding. The staff comprises industrial product designers, user experience designers, electrotechnicians, a quality adviser, a manager, and a coordinator who is also an academic researcher. The staff collaborates with individuals who identify a problem, such as patients, to develop technical innovations that address the experienced problem. Cocreation is embedded throughout all phases of the design thinking process. Depending on the situation, patients either visit the makerspace studio or designers meet them at the hospital ward. This makerspace aims to deliver functional prototypes that comply with relevant regulatory requirements. It operates in accordance with the Medical Device Regulation and produces medical devices for in-house use under the hospital’s quality management system, which includes preparing required documentation such as an innovation justification statement, a statement of requirements, and risk analyses. The makerspace has successfully produced over 30 innovative products for use in daily practice. The makerspace staff proactively disseminates their knowledge and products within the hospital and beyond.

**Figure 1.**
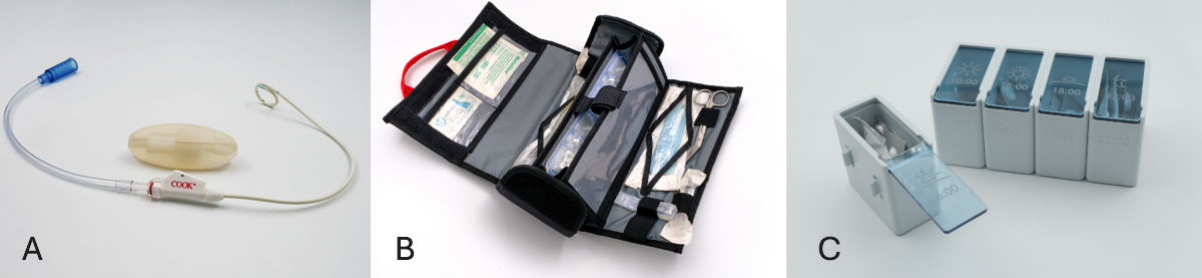
Examples of technical innovations developed in cocreation with patients within the makerspace: (A) Silicone cover kidney drain, (B) Emergency bag tracheostomy tube, and (C) Pillbox for hospitalized patients.

Patients or carers typically approach the makerspace with a specific problem and cocreate with the makerspace staff to develop a solution. Examples of innovations include a silicone cover designed to fit the rigid connection piece of a kidney drain, reducing discomfort during daily activities and sleep (Figure 1A); an easily accessible emergency bag to support rapid response when a child’s tracheostomy tube becomes obstructed (Figure 1B); a pillbox enabling hospitalized patients to manage their own medication while allowing nurses to maintain oversight of medication administration (Figure 1C); a waterproof shower cape for patients with a Port-a-Cath for intravenous chemotherapy; and a bedside table that can be positioned over the bed to facilitate play, homework, or family meals.

### Population and Recruitment

To gain insight into the perspectives of both designers and end users, a heterogeneous group of designers and patients was selected using purposive sampling [27]. The first perspective, referred to as the “designer” perspective, included makerspace staff such as industrial product designers and user experience designers. The second, the “patient” perspective, included individuals involved in cocreating innovations addressing problems experienced by patients. This group included patients and representatives such as informal caregivers (eg, parents in the case of young children) and professionals who supported patients during the cocreation process and were able to describe their experiences with patients from their perspective. Eligible participants were individuals who had participated in a collaborative project with the hospital’s makerspace to develop an innovation addressing issues experienced by patients and who represented either the designer or patient perspective. Designers who had no direct interaction with patients during collaborative projects were excluded. Additionally, patients under the age of 18 years or those who were hospitalized and in a delicate condition at the time of the study were excluded, as they were considered unable to participate adequately in an interview or participation could impose an undue burden.

Eligible designers and patients (or their representatives) were identified through the makerspace’s network of previous collaborations. For recruitment, individuals who had cocreated innovations at the makerspace, preferably pairs who had collaborated on projects, were approached and informed about the study by the makerspace’s coordinator, who is also senior researcher OH. Eligible participants had to actively make contact with the study’s first researcher, JVDG, via email. Once contact was initiated, they were sent the participant information letter and informed consent form (ICF). JVDG then scheduled an interview with those who had confirmed their willingness to participate.

### Data Collection

Researcher JVDG conducted individual face-to-face and online interviews to collect the data. With a background in nursing and no affiliation with the hospital, JVDG brought relevant experience while maintaining impartiality. A semistructured interview guide was developed, based on the Sunnybrook team–based competencies [28] and the design thinking [17,29] approach to innovation development (Multimedia Appendix 1). The guide was chronologically structured around the phases of innovation: inspiration, ideation, and implementation. A single interview guide was developed, reflecting the equal role of patients within interprofessional teams as emphasized in the Sunnybrook model [28]. The content and language of the interview guide were reviewed by an external expert in patient-driven innovation and senior researchers OH and TVH. It was pilot-tested in 1 interview and subsequently refined to ensure relevance and clarity. After the first 4 interviews, the guide was further adjusted based on emerging insights.

At the beginning of each interview, participants completed a brief demographic form, which included categorical questions about age, innovation experience, and professional perspective based on their employment. All interviews were audio-recorded. A constant comparison method was applied during data collection and analysis, leading to iterative adjustments to both the interview guide and the codebook (for further details, see the “Data Analysis” section). Data collection stopped once data saturation was confirmed.

### Data Analysis

Researcher JVDG transcribed the interviews verbatim. Data were analyzed using a thematic approach proposed by Boeije and supported by NVivo (version 14; Lumivero) qualitative research software [30]. This approach consists of 2 phases: segmenting and reassembling. In the segmenting phase, JVDG reread the transcripts and inductively assigned codes to relevant text fragments. In vivo coding was applied to the first 7 interviews. During axial coding, these initial codes were grouped into broader categories, resulting in a preliminary codebook with broader codes, which was then used to code the remaining interviews. This phase was also carried out by JVDG. Before the reassembling phase, JVDG created a mind map to become familiar with the coded data. In the reassembling phase, the axial codes were interpreted and discussed with senior researchers OH and TVH, leading to the development of conceptual themes and categories. An independent researcher with expertise in qualitative research reviewed the conceptual themes, whereupon JVDG established the final themes. Consensus on the final themes was reached among researchers OH, TVH, and JVDG. Representative quotes and descriptions of themes and categories were derived from the original Dutch data and translated into English.

### Study Rigor

The study’s credibility was enhanced through member checking and researcher triangulation. Participants received a summary of the key points from their interviews and were invited to provide feedback. The feedback received was incorporated into the data to ensure alignment with participants’ perceptions. Researcher triangulation was achieved through weekly meetings between researchers OH, TVH, and JVDG to discuss and monitor the study’s progress. Additionally, 2 external researchers—1 with expertise in patient-driven innovation and 1 in qualitative research—were consulted during various phases of the study. To enhance confirmability, a digital logbook containing observational, methodological, and analytical memos was maintained throughout the research process [31].

### Ethical Considerations

The study adhered to the principles outlined in the Declaration of Helsinki [32], as well as the European [33] and Dutch codes of conduct [34]. Participants received verbal information about the research and were sent an information letter and ICF via email by the researcher JVDG. All participants signed the ICF prior to the start of the interview. Data confidentiality was ensured through pseudonymization. The study was reviewed and approved by the medical ethics review committee of the Erasmus Medical Center for a nonmedical scientific research application (MEC-2024‐0019).

## Results

### Overview

In total, 12 individuals participated: 6 with the designer’s perspective and 6 with the patient’s perspective (Table 1). The patient perspective was represented by 2 patients, 2 parents, 1 staff member of the Children’s Advisory Council who helped patients during the cocreation with the makerspace, and 1 nurse who cocreated with patients and the makerspace to develop an innovation to enhance patients’ self-sufficiency in medication administration. All invited eligible individuals agreed to participate. Among these 12 participants, 4 designer-patient pairs were included. From the 8 cocreated innovations discussed in the interviews, 4 innovations were developed for children, 3 innovations for adults, and 1 innovation was primarily developed for a child’s caregiver. Most of the participants were equally distributed between the age ranges of 18 to 35 and 36 to 50 years. All designers had more than 3 years of previous experience in innovation projects; patients mostly had none or up to 3 years of such experience.

**Table 1.** Demographics of participants (N=12).

Characteristics and categories	Participants, n (%)
Age range (years)	
18-35	5 (42)
36‐50	5 (42)
51‐65	1 (8)
>65	1 (8)
Previous experience with innovation (y)	
None	2 (17)
<0.5	0 (0)
0.5‐1	1 (8)
1‐3	2 (17)
>3	7 (58)
Perspective	
Industrial product designer	5 (42)
User experience designer	1 (8)
Parent	2 (17)
Patient	2 (17)
Staff member of Children’s Advisory Council	1 (8)
Nurse	1 (8)

After 8 interviews, no new categories emerged, indicating data saturation. To confirm and assess the identified themes, 4 additional interviews were undertaken. The interviews lasted an average of 51 (SD 19) minutes, with durations ranging from 25 to 90 minutes, depending largely on the participant’s level of involvement in the cocreation. Three interviews were conducted online for convenience, while 9 interviews took place at the hospital. One interview included the patient’s partner. Member-check feedback was obtained from 10 participants, all of whom agreed with the summary made by JVDG. Two minor corrections were made based on their feedback. Despite an email reminder, responses from 2 participants were not received.

**Figure 2.**
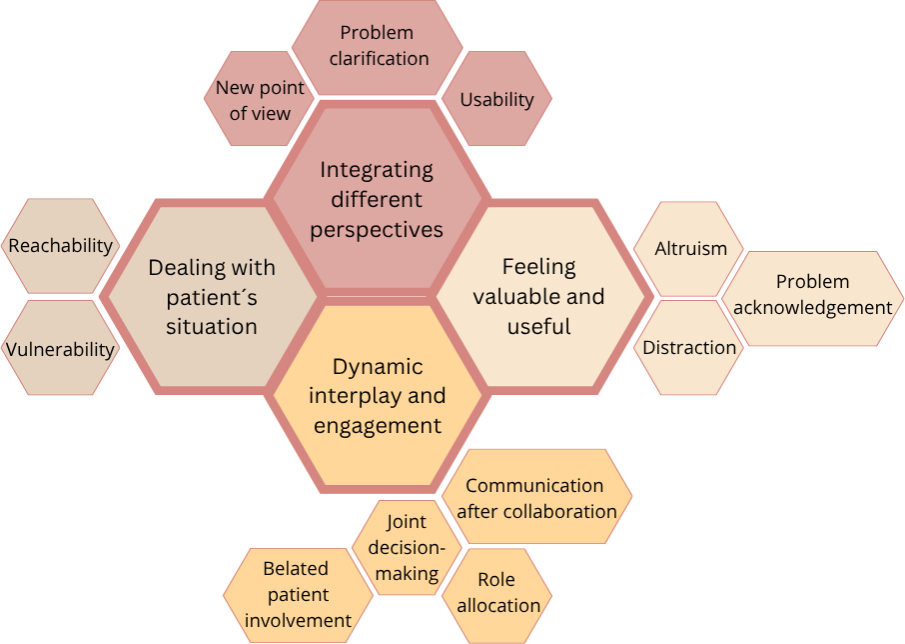
Four themes (represented by the larger hexagons) and 12 associated subthemes regarding the experiences of designers and (carers of) patients cocreating in a hospital’s makerspace.

The following themes emerged: dealing with the patient’s situation, integrating different perspectives, feeling valuable and useful, and dynamic interplay and engagement (Figure 2).

### Theme: Dealing With the Patient’s Situation

The circumstances of patients differ significantly from the everyday experiences of healthy individuals. This theme explores the challenges and implications that these differences create for patients and designers during their collaboration.

#### Vulnerability Due to the Patient’s Health

Participants noted that a patient’s compromised health often limits their ability to engage fully in the innovation process, although they may have wanted otherwise. Illness brings competing concerns such as fatigue. Designers—particularly younger ones—are often aware of this vulnerability. For many, cocreation with patients is a new experience, and they may hesitate to impose an undue burden on patients. Some designers struggle with knowing what is appropriate to ask or expect from patients under these circumstances:

*I was sometimes a bit ashamed to ask things, because I felt like the person would then literally have to think back to such a moment [traumatic experience] you know, just to answer my question*.

[P3, user experience designer]

#### Reachability

Participants suggested that maintaining low-key contact and close involvement increases the patient’s reachability. However, they also noted that a patient’s reachability is often limited by their health circumstances. First, rapid changes in the patient’s condition can lead to unexpected transfers to another ward or home. Designers found it challenging to reach a patient after discharge:

*It was by chance that [child’s name] was admitted, so I could just walk back and forth, but in the future, I would like to be more physically available. If someone says, “come and have a look,” I would like to be able to do that, and now that’s not possible.*

[P12, parent]

Second, designers were hesitant to visit patients on their wards because they do not interact with patients on a daily basis, and extra effort was needed. This was perceived as a barrier. Once on the ward, some designers felt uncomfortable or rushed due to the ward’s often hectic environment:

*But that is just how the intensive care unit is. It is hectic. You don’t know at what time someone will come to the bedside [of the patient]. So that was for me a challenge.*

[P3, user experience designer]

Additionally, the makerspace is not yet well-known among patients. This lack of knowledge has made it difficult for a patient to access the makerspace. In many collaborations, intermediaries often facilitate contact between designers and patients:

*Yeah well, I was lucky my nurse knew [industrial product designer 1], otherwise I would never have come up with it.*

[P4, patient]

Patients emphasized that, for both parties, it simply comes down to asking. They value their autonomy in decision-making and felt encouraged to express whether or not they wished to participate in a cocreation.

One less experienced designer wondered why she had not collected the patient’s contact information during their hospital stay, which would have allowed for continued collaboration after discharge.

### Theme: Integrating Different Perspectives

This theme explores the value of incorporating multiple perspectives and the challenges that may arise as a result.

#### New Point of View

Incorporating multiple perspectives provides a range of viewpoints during the innovation project. Both designers and patients noted that differing perspectives can generate fresh insights into the problem or proposed solution. Both parties felt as though the other party had been given carte blanche:

*Sometimes, you just have to give people carte blanche because you're already too involved. I had my own bag [tracheostomy tube emergency bag], so I was too much into that concept. You know; this is how we do it. So, I would just show that. But that is not helpful at all, because sometimes you have to start from scratch*.

[P7, parent]

*No, as blank as possible, without any prior knowledge that you just shout what is possible, what you think is possible. That’s just beautiful [...]. And then someone else who knows more about it can say, well, this might be possible, and this might not be. I wish they did that more often, [...]. No idea is crazy enough, so to speak*.

[P1, industrial product designer]

#### Problem Clarification and Usability

Designers emphasized the importance of the patient’s perspective in clearly defining the problem. Participants also agreed that integrating multiple perspectives had a positive impact on the usability of the innovation in practice. Some noted that when an innovation is not adopted in practice, it is often because the needs of all stakeholders were not adequately identified:

*And it turned out they [patients] did not like it at all. And then I looked back, and I thought, yes, I can understand that, because we didn't involve them at all in that initial new design. We did ask those parents; what do you prefer? What is important to you? What should come out of it for you? We actually completely forgot to include the patient*.

[P6, staff member of Children’s Advisory Council]

However, 1 designer pointed out that the inclusion of multiple perspectives, such as a project involving a patient, parent, and nurse, could also cause interference. The differing needs made it harder to clearly define the core problem, and the designer felt that the patient’s needs were overshadowed:

*And that is what I mean by those different perspectives: I get a lot of information but then I wonder, how is it from the child’s point of view? It was not clear to me which pain points the child was really experiencing*.

[P3, user experience designer]

### Theme: Feeling Valuable and Useful

This theme captures how cocreation gave both designers and patients a sense of contributing to something valuable and useful.

#### Altruism

All participants expressed a strong motivation to contribute to the improvement of health care, even if they did not benefit directly from the outcomes. They desired to help solve a problem and do something for others:

*I always like to participate in these kinds of experiments. To make people’s lives more comfortable.*

[P9, patient]

*Well, someone who is sick, who has a problem and addresses this to ultimately help others sort of, with that approach, running such a project, I think is really great*.

[P1, industrial product designer]

#### Distraction

For patients, participation also served as a distraction from their medical situation. It provided them with a sense of activity and usefulness, allowing them to contribute even in the midst of an unpleasant situation, “And then … yeah, I was bored to death anyway, so I thought yes let’s do it” (P4, patient).

#### Problem Acknowledgment

Both designers and patients felt that the innovation process validated the underlying problem. They appreciated that it was taken seriously and actively addressed. This acknowledgment gave participants a sense that they did not have to simply accept problems or settle for inadequate solutions:

*Once I had a parent standing in front of me, almost in tears, and she said something like: I think it is really wonderful that there are people who think about those small, seemingly insignificant things, but it makes life easier for such people*.

[P10, industrial product designer]

### Theme: Dynamic Interplay and Engagement

#### General Outline

This theme explores the depth of cocreation throughout the innovation process, with a focus on the interplay between designers and patients. The interviews revealed instances where the perceived level of cocreation did not align with the patient’s actual involvement.

Participants highlighted that cocreation is highly contextual, varying depending on factors such as the stage of the innovation process and the patient’s role. The level of patient involvement often depended on their personal interest, enthusiasm, or health condition:

*Genuinely working together, yeah, it’s possible, but it’s difficult. And I think it really depends on the patient or depends on the setting and context*.

[P2, industrial product designer]

#### Role Allocation

Some participants wondered if the cocreation could be considered truly collaborative, as patients were not fully involved throughout the entire design process. Designers often took a leading role, with patients only providing feedback on developed concepts:

*It’s constantly; you go and test something, you receive feedback, you go and redesign it, and you go and test it again.*

[P11, industrial product designer]

One designer suggested that genuine cocreation would involve patients actively contributing ideas and helping to design prototypes—essentially taking on a co-designer role. However, the designer also questioned whether this expectation was realistic for patients. Many patients expressed that their level of involvement—whether throughout the entire process or within a single phase—felt appropriate. Contributing to just one part of the cocreation required less time and energy, yet still gave them a sense of satisfaction, particularly knowing that their input could benefit others:

*If I had been in my full power, I might have done more exploring or for example thought what can we do with this, you know? [...] But now I had so much on my mind, I left it as it was. But I enjoyed it, though.*

[P4, patient]

#### Belated Patient Involvement

Designers expressed concerns about burdening patients, which led them to consider involving patients only in a single phase of the design process. They also noted that excluding patients early on benefited their individual creative process, enabling them to freely explore multiple design options. Often, designers developed a physical prototype before involving patients, as they believed that patients provided more useful feedback on tangible products than on abstract ideas on paper:

*Because if you start describing it [idea of concept of the innovation] then they might also have questions something like “yes but how about that?” So, seeing a model or something may lead also to other ideas*.

[P11, industrial product designer]

Designers highlighted the risk of making unintentional assumptions during the process, which can impact the final design and limit the ability to make changes later. Two designers expressed that they would have preferred to involve the patient earlier in the process. Consequently, participants confirmed that valuable information related to the problem or solution could have been overlooked by excluding patients’ perspectives in the early phases:

*And then you have to take them along from the very beginning, not asking them to test it when you already have developed the [innovation], because that [innovation] might work, but the question is whether that is the best fitting innovation for those parents, because maybe there was something much better*.

[P6, staff member of Children’s Advisory Council]

#### Joint Decision-Making

Participants indicated that decisions were largely made collaboratively, which they perceived as pleasant and fostered a sense of equality. This approach facilitated open communication and contributed to a good atmosphere in the makerspace. Patients felt free to express their opinions about the design. While joint decision-making sometimes led to shared disappointment when a design fell short, all participants ultimately reflected positively on the cocreation and were satisfied with the final innovation.

*It was always a very open conversation, and yeah, we, she was like the designer, and we were the ones presenting the problem, so to speak, but we could really meet each other halfway*.

[P5, patient’s perspective represented by nurse]

#### Communication After Collaboration

Participants highlighted the importance of letting patients know—as a sign of appreciation—how their feedback was incorporated. However, many patients said that they missed such communication and wondered how the innovation was further developed.

*After that, I didn't hear how it ended, and I find that a shame, do you understand? So, then you think, well, he said maybe we'll apply it throughout the whole hospital during that treatment. It would be nice for those who in this case participated to know what happens next to it.*

P9, patient]

One patient discovered that the innovation had not been further developed, and they felt that the innovation was “just” a project that had been shelved. Several participants indeed noted that innovations are sometimes not further developed due to a lack of interest and funding.

## Discussion

### Principal Results

This study focused on the cocreation between designers and patients (or their carers), exploring their experiences throughout the innovation process. Four key themes were identified. The first theme, dealing with the patient’s situation, highlighted challenges related to patients’ vulnerability and limited availability due to their condition. The second theme, integrating different perspectives, emphasized the value of incorporating diverse viewpoints, which helped clarify the problem. However, it also revealed that differing perspectives could complicate problem definition. The third theme, feeling useful and valuable, described how participation fostered a sense of meaningful contribution among patients, offering both a positive distraction and a sense of validation regarding their experienced problem.

The fourth theme, dynamic interplay and engagement, focused on the nature of the interactions within the cocreation. During the interviews and analysis, questions arose about how to distinguish genuine cocreation from surface-level involvement. Despite this, participants generally viewed each other as equal partners. Key aspects within this theme included role allocation, the extent of patient involvement, decision-making, and communication. While existing literature provides limited insights into patient-designer interaction within the cocreation process in hospital-based makerspaces, this study provides a better understanding from both perspectives.

### Strengths and Limitations

A strength of this study was the high quality of the data collected using an interview guide based on the Sunnybrook team–based competencies in combination with the design thinking model. Additionally, the use of the constant comparison technique allowed for iterative adjustments to the interview guide, enabling interviews to more effectively address relevant topics. This approach facilitated deeper exploration and assessment of conceptual themes, ultimately leading to data saturation. The high response rate in the member check procedure confirmed that the data accurately reflected participants’ experiences, thereby increasing the study’s credibility. Another strength was the inclusion of designer-patient cocreation pairs across diverse innovation projects, each with varying levels of patient involvement. This diversity provided detailed insights into how different perspectives interpreted the same collaborative experience.

A limitation of this study is that it was conducted at a single hospital and with a relatively small population of eligible participants. However, for each cocreation between designers and patients within the makerspace, all eligible individuals were contacted. Both perspectives were ultimately represented by 6 participants. Literature suggests that data saturation in heterogeneous samples is typically reached between 9 and 16 interviews, depending on the sample characteristics [35]. We observed a consistent overlap in overarching categories after 8 interviews. Therefore, we consider our sample size to be appropriate for this study.

The patient perspective was represented by 2 patients and 4 individuals who were not patients themselves: 2 parents, a nurse, and a member of the children’s advisory board. Because half of the cocreated innovations focused on children, some findings may be slightly oriented toward pediatric contexts. While these participants drew on their own experiences and their interpretations of patients’ experiences during cocreation, it remains uncertain how their inclusion may have shaped the patient perspective described in this study. Certain overarching themes, such as dealing with the patient’s situation, may initially appear misaligned with a strictly patient-focused view. However, emotional and physical vulnerability were also central to the parents’ experiences and therefore remained relevant to our analysis.

Importantly, the nonpatient participants contributed substantial experiential knowledge based on their involvement in cocreation processes with patients and the makerspace. Given that our findings are consistent with existing literature from comparable settings, we consider these complementary perspectives as meaningful additions to the patient perspective rather than a distortion of it.

Another limitation is the potential for recall bias, as some projects took place several years ago. While fading memories may have influenced participants’ perceptions, many reported that the interview process helped revive their recollections.

### Comparison With Prior Work

Our study highlights how incorporating the patient’s perspective can enhance the understanding of user needs, leading to better problem clarification and greater usability of innovations. These findings support previous research on patient involvement in service innovation, as well as literature emphasizing broader stakeholder engagement in health care [19,36-38]. However, our study also sheds light on the complexity of developing technical innovations that involve various end users, including hospitalized patients. Identifying and addressing the diverse needs of stakeholders—such as patients, nurses, infection prevention specialists, and manufacturers—while also complying with existing regulations poses a significant challenge.

Within the makerspace of this study, the average time between the start of development and the implementation of the innovation is 19 months, ranging from 6 to 48 months. This extended timeline often exceeds the duration of hospital stays for patients and is further complicated by the high turnover of patients, making it difficult for designers to reach and involve patients throughout the development process, as observed in our study. Compromised reachability due to these short admissions could explain the belated involvement of patients.

The vulnerability experienced by designers and patients is likewise seen in the literature, where patients’ physical and mental conditions influence their preferred level of involvement [39,40]. In a qualitative study by Rowland et al [38] exploring the patient’s perspective, vulnerability was identified as an essential element of the patient experience, contributing to a unique form of embodied knowledge. While participants in our study acknowledged the value of the patient perspective, this same vulnerability often led to hesitation and hindered involvement. This finding offers new insights into how patient vulnerability can impact cocreation and innovation development within a makerspace context. A mixed methods study on motives for involvement in service innovation found that patient engagement fostered feelings of accomplishment and was often driven by a desire to give back, as well as by social connection and enjoyment [41]. These motivations closely align with the theme of feeling valuable identified in our study.

A systematic review on service innovation highlighted the gap between the intention to involve patients and the practical challenges of doing so—particularly in joint decision-making processes [42]. The review emphasized that care providers require proper attitudes, skills, and a deeper understanding of patients to facilitate meaningful involvement. Our finding about the lack of communication with patients after the collaboration has not been explicitly reported in previous literature. However, a co-design framework for health care innovation includes a final step of revisiting co-design participants to share the outcomes of the project [23]. This step appears to have been missed by the patients in our study.

Delayed patient involvement, as observed in our study, limited the optimal use of patient contributions. Previous literature similarly indicates that stakeholder involvement often occurs primarily during the front-end prototyping phase, rather than during the earlier inspiration phase of design thinking or consistently across all phases of development [20,43,44]. A qualitative study identified 17 strategies for stakeholder involvement, 1 of which involved real-time modification of prototypes in collaboration with stakeholders [43]. However, this strategy was found to be underused, which aligns with our findings regarding limited patient participation across all development phases.

### Implications for Practice

This study underscores the importance of involving patients and their carers in the development of health care innovations. Their unique perspectives are highly valuable, particularly for innovations related to patient care. The findings offer insights into how patients and designers collaborate in innovation development and provide practical guidance for hospital-based makerspaces seeking to strengthen cocreation with patients (Textbox 1). Makerspaces should actively evaluate their cocreations and ensure that cocreation experiences are systematically assessed through patient feedback.

While this study explored the makerspace’s previous patient cocreations, it raises the question of how to increase the frequency of patient cocreation. Patients in our study were previously unaware of the existence of the makerspace. Therefore, makerspaces should research strategies to enhance their visibility and accessibility to patients. It is crucial for patients who experience troubling issues to know that a makerspace is available to help them find solutions. Additionally, makerspaces should strive to involve the patients’ perspectives in a broader range of projects, beyond those directly related to patient-experienced issues. By doing so, innovations will be better aligned with patients’ needs and experiences, ultimately leading to more effective and user-centered health care solutions.

Textbox 1. **Practical tips for hospital-based makerspaces regarding cocreation with patients.**

The tips are as follows:

- Let patients speak for themselves. Respect patient autonomy in decision-making. Do not hesitate to initiate contact out of concern it may burden the patient. Participation can evoke positive emotions.
- Involve patients early. Engage patients from the problem formulation phase, as they can offer valuable clarification. When their condition allows it, involve them throughout all development phases.
- Maintain communication after collaboration. Keep patients informed about the further development of the innovation. Continued communication reinforces their sense of involvement and appreciation.
- Gather ongoing feedback. Regularly collect feedback from patients on their experiences and suggestions. This helps to refine the cocreation process and ensures that future collaborations are more inclusive and effective.

### Conclusions

This study explores the cocreation between designers and patients (or their carers) from both perspectives and offers valuable insights into the cocreation process of patient-initiated technical innovations within a hospital-based makerspace. While cocreation is widely perceived as valuable in innovation development, its success is influenced by the patient’s individual situation and their level of involvement. To strengthen future cocreation, makerspaces may benefit from applying practical strategies to effectively involve patients.

## Supplementary material

10.2196/85926 Multimedia Appendix 1 Interview guide.

10.2196/85926 Checklist 1 COREQ checklist.

## Abbreviations

COREQ: Consolidated Criteria for Reporting Qualitative Research
ICF: informed consent form
