# Supplementary material for: Patient and Designer Collaboration in Cocreating Technical Innovations in a Hospital-Based Makerspace: Qualitative Interview Study
Source: JMIR Hum Factors. 2026 May 20;13:e85926. doi: 10.2196/85926 (PMC13189569; doi:10.2196/85926)
Supplement: Multimedia Appendix 1 [file humanfactors-v13-e85926-s001.docx]

Interview guide

**Introduction**

- Welcome
- Explaining purpose of study and the interview: "The purpose of the study is to increase the patient involvement in innovation development in the future. The purpose of this interview is to learn more about how you experienced the collaboration during the development of the innovation [innovation name] with [Create4Care / patient involved]. You were involved there from your role as [(family of) patient/ professional occupation]."
- Refer to the informed consent, audio recording, possibility to quit.
- Explain interview structure: collaboration following the design thinking steps as a reminder and eventually a general review on the collaboration.

**First**: “Before we dive deeper into the collaboration, I would like to know how the collaboration was initiated”

- How did you come to Create4Care?
  - to what extent were you already familiar with Create4Care?
- What made you decide to participate? {Examine the underlying motivation to design an innovation through an active collaboration with patients}
  - What expectations did you have at the beginning of the collaboration?
- Could you tell me about your role in the collaboration?
  - Was it clear to you from the beginning what was expected of you?
- {examine at what phase the patient and designer both were involved}

How far was the innovation developed when you were involved in the collaboration? When applicable with designer: when was the patient involved in the innovation process?

*{Give a brief summary of how the participant was involved in the collaboration. Check to see if this conclusion is true with the participant and if there are any important additions}.*

*{Start at the stage where the participant was involved in the collaboration with patient/Create4Care. Provide the explanation using the illustration of the innovation process. This means that not every phase is questioned with everyone during the interview}.*

**Innovation development phases**

**Inspiration**: In this phase, the aim is to gather as much information as possible about the problem to outline where the needs are. Afterwards, all information is compiled to describe the core problem.

- Do you remember the moment when you thought: ‘Yes, this is the problem we need to tackle!’?
  - What did that moment look like?
  - What was the problem?
  - Where or from whom did the problem/need for a solution originate?
- How was this problem explored in depth?
  - Did you approach different stakeholders during the collaboration?
- Can you tell me more about what you did in this phase from your perspective as *[(family of) patient / designer]*?
  - Do you feel that you were able to contribute to gathering information and identifying the problem? Can you explain/give an example?
- Facilitators: Looking back on this part of the collaboration, what went well during the collaboration with *[patient / Create4Care]*?
- Barriers: Were there things you encountered that didn't go well or made you feel uncomfortable?
  - Was this addressed during the collaboration? How was it handled? Did you have the feeling it was resolved?
  - How could this have been prevented?

**Ideation**: In this phase, as many solutions as possible are thought off and designed for the core problem, considering all the different people involved with the problem from the first phase.

- Can you tell me more about what you did in this phase from your perspective as *[(family of) patient / designer]*?
- How were ideas about designs/solutions established and shared?
  - Were you asked if you had an idea for a solution?
  - Was anything done with the feedback you provided?
  - Did everyone come to an agreement on which ideas/solutions could be further developed? Can you describe how this process went?
- Facilitators: Looking back on this part of the collaboration regarding brainstorming solutions, what went well during the collaboration with [patient / Create4Care]?
- Barriers: Were there things you encountered that didn't go well or made you feel uncomfortable?
  - Was this addressed during the collaboration? How was it handled? Did you have the feeling it was resolved?
  - How could this have been prevented?

**Implementation**: In this phase, the design of a test version is tested and prepared for practical use and implementation.

- Was a test version of the solution created and tested?
- If so, did you help during the testing of that test version?
  - What did you help with (example)?
  - Was anything done with the feedback you provided?
- ALWAYS ASK NEXT QUESTION*: {Provide a description of the final version/result of the innovation}.* Is this (so far) the result of the innovation?
  - What do you think of the result?
- Facilitators: Looking back on this part of the collaboration, what helped you during the collaboration with [patient / Create4Care]? What went well?
- Barriers: Were there things you encountered that didn't go well or made you feel uncomfortable?
  - Was this addressed during the collaboration? How was it handled? Did you have the feeling it was resolved?
  - How could this have been prevented?

**Last**: We have looked at each phase to see what happened and what went well and not. Looking back on the entire collaboration:

**Questions for the patient:**

- How did you find your involvement in the collaboration?
- How did your involvement contribute to the development of the innovation *[name of innovation]? Did it add value?*
- What do you think of the level of patient involvement?
  - *More/less? Earlier or later in the process?*
- What are your thoughts on how patients can be involved in innovation developments by Create4Care?
- How was the communication during the collaboration between you and Create4Care?
  - Was it easy to participate in group discussions?
- If you were to engage in a similar collaboration for developing an innovation in the future;
  - What would you do the same?
  - What would you do differently?

**Questions for the professional:**

- How did you find the inclusion of the patient/patient perspective in the collaboration?
  - Did it contribute to the development? Was there something you, as a designer, hadn't thought of? *Did it add value?*
- What do you think of the level of patient involvement?
  - *More/less? Earlier or later in the process?*
- What are your thoughts on how patients can be involved in innovation developments by Create4Care?
- How was the communication during the collaboration between you and the patient?
  - Was it easy to involve the patient in the discussions?
- If you were to engage in a similar collaboration with a patient in the future;
  - What would you do the same in the next collaboration with a patient?
  - What would you do differently?

*{Indicate that you have reached the end of the interview.}*

**Ending:**

- Review the interview experience: How did you find this conversation? In your opinion, did I forget to ask anything, or is there something that hasn't been covered that you would like to mention?
- Thank the participant for his/her time.
- Indicate that a summary will be sent within three weeks.


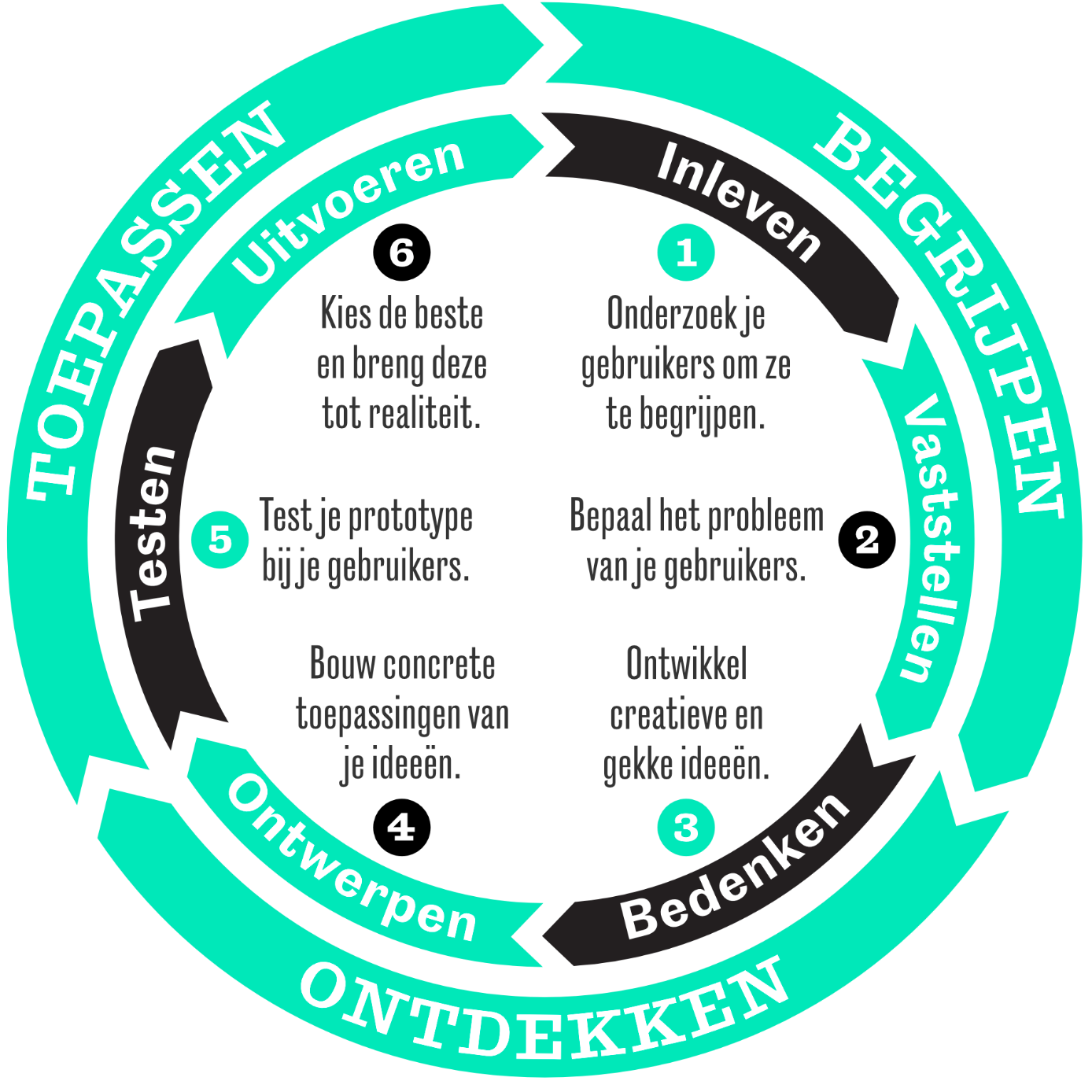


Figure 1: Graphic overview of development process by Manufesta^1^

1: Manufesta. Design thinking: elk verander proces is een designproces [Internet]. [Geciteerd 2023-nov-09]. Beschikbaar van: https://manufesta.nl/blog/design-thinking-elk-veranderproces-is-een-designproces
